# Utilization of individual components of enhanced recovery after surgery (ERAS) protocol improves post-operative outcomes in adolescent idiopathic scoliosis: a blueprint for progressive adoption of ERAS

**DOI:** 10.1007/s43390-023-00706-w

**Published:** 2023-05-26

**Authors:** David E. Lebel, Masayoshi Machida, Robert Koucheki, Fiona Campbell, Natasha Bath, Martin Koyle, Danielle Ruskin, David Levin, Sarah Brennenstuhl, Jennifer Stinson

**Affiliations:** 1 Division of Orthopaedic Surgery, The Hospital for Sick Children, Toronto, ON Canada; 2 Division of Orthopaedic Surgery, Department of Surgery, University of Toronto, Toronto, Canada; 3 Institute of Biomedical Engineering, University of Toronto, Toronto, Canada; 4 Division of Urology, The Hospital for Sick Children, Toronto, Canada; 5 Department of Anesthesia and Pain Medicine, The Hospital for Sick Children, Toronto, Canada

**Keywords:** Enhanced recovery after surgery, ERAS, Rapid recovery pathway, RRP, Idiopathic scoliosis, Spine surgery

## Abstract

**Purpose:**

Enhanced recovery after surgery [ERAS] is an approach for standardization of perioperative care aimed at improving patient outcomes. The primary aim of this study was to determine if length of stay (LOS) differed by protocol type (ERAS vs. non-ERAS [N-ERAS]) in patients undergoing surgery for adolescent idiopathic scoliosis (AIS).

**Methods:**

A retrospective cohort study was conducted. Patient characteristics were collected and compared between groups. Differences in LOS were assessed using regression adjusting for age, sex, BMI, pre-surgical Cobb angle, levels fused and year of surgery.

**Results:**

Fifty nine ERAS patients were compared to 81 N-ERAS patients. Patients were comparable in their baseline characteristics. Median LOS was 3 days (IQR = 3–4) for the ERAS group, compared to 5 days (IQR = 4–5) for the N-ERAS group (*p* < 0.001). The ERAS group had a significantly lower adjusted rate of stay (RR = 0.75; 95% CI = 0.62–0.92). The ERAS group had significantly lower average pain on post-operative days 0 (least-squares-mean [LSM] 2.66 vs. 4.41, *p* < 0.001), POD1 (LSM 3.12 vs. 4.48, *p* < 0.001) and POD5 (LSM 2.84 vs. 4.42, *p* = 0.035). The ERAS group had lower opioid consumption (*p* < 0.001). LOS was predicted by the number of protocol elements received; those receiving two (RR = 1.54 95% CI = 1.05–2.24), one (RR = 1.49; 95% CI = 1.09–2.03) or none (RR = 1.60, 95% CI = 1.21–2.13) had significantly longer rates of stay than those receiving all four.

**Conclusion:**

Adoption of modified ERAS-based protocol for patients undergoing PSF for AIS led to significant reduction in LOS, average pain scores, and opioid consumption.

**Supplementary Information:**

The online version contains supplementary material available at 10.1007/s43390-023-00706-w.

## Introduction

Enhanced recovery after surgery (ERAS) is an approach for standardization of perioperative care aimed to reduce the stress response and retain anabolic homeostasis. ERAS implementation has been shown to significantly improve process and patient outcomes across several surgical domains, including in general surgery, gynecology, and urology [1].

Posterior spine fusion (PSF) for adolescent idiopathic scoliosis (AIS) is a common surgery within pediatric orthopedic practices. The large surgical stress, high degree of surgical complexity, propensity for large volume blood loss, impact of post-operative pain on capacity to comply with physiotherapy, and the need for multidisciplinary collaboration makes this surgical pathology and patient group a strong candidate to benefit from ERAS protocol-based treatments.

Reports of successful ERAS implemented in orthopedic surgery were first in arthroplasty, and later in spine surgery [2, 3]. It has been shown that ERAS pathways lead to decrease in length of stay (LOS), cost, and pain scores among patients undergoing major spine surgeries without increase in readmission rates [4, 5]. Similarly, a rapid recovery after surgery (RRAS) approach has been evaluated in PSF for AIS in several studies [6–11]. Those RRAS initiatives demonstrated that introduction of protocol reduced LOS and costs of treatment. Recently, an ERAS-based study on operated patients with AIS revealed differences in LOS and post-operative pain comparing two different institutions within different health care systems [10].

To standardize care of AIS patients undergoing PSF at our tertiary free-standing pediatric hospital, a four element ERAS protocol was established in 2018. We defined the ERAS elements based on the ‘ERAS Society Guideline Elements for Colonic Resection’ described by Ljungqvist et al. [1], We chose the elements that were felt to best fit our institutional practices and this specific patient population. This approach is in keeping with most of the published orthopedic literature regarding ERAS implementation in PSF. The specific protocol elements that were introduced included: (1) neuraxial opioid analgesia and (2) the use of non-steroidal anti-inflammatory drugs (NSAID); (3) early mobilization; (4) early urinary catheter removal. The decision to treat patients using this ERAS protocol was largely surgeon driven, with some surgeons choosing to adopt either none, or only some elements of the care bundle. This created an opportunity to study the outcomes related to the compliance with the ERAS protocol.

We present a retrospective comparative review of patients that were treated with PSF for AIS at the same institution during the same time period with different degrees of adherence to the ERAS protocols. The primary objective of the study was to determine if there were differences in LOS according to protocol type. The secondary objectives were to: (1) assess differences in opioid consumption and pain scores over time according to protocol type and (2) identify predictors of LOS. It was hypothesized that modified ERAS introduction will shorten LOS and reduce both pain scores and opioid consumption.

## Methods

This study is a retrospective chart review of consecutive patients with AIS treated in a single institution with PSF and instrumentation between August 2018 and December 2020. Inclusion criteria were age 10–18 years, diagnosis of idiopathic scoliosis, and undergoing PSF. Exclusion criterion was non-idiopathic scoliosis. Patients were divided into two groups based on treatment protocol: ERAS-based group (ERAS) and Non-ERAS-based group (N-ERAS) (Table 1). To be included in the ERAS group patients had to be treated with multimodal analgesia based on intrathecal morphine (ITM) and NSAIDs. All those in the ERAS group were treated by a single physician. Post-operative chart review was performed by a nurse practitioner and research associate. The data were collected using the REDCap^®^ platform. Follow up was completed up to 12 months after surgery to account for late re-admissions. The study received research ethics board approval from the host institution.

**Table 1:** ERAS vs N-ERAS protocols

ERAS	N-ERAS
Multimodal analgesia based on ITM	Multimodal analgesia based on 48 h of IV PCA
NSAIDS	Urinary catheter for 48 h following stop of IV PCA
Urinary catheter removal on day 1, early morning	Ambulation starts on day 1 to seat, walk on day 2
Early ambulation–seat day 0, Walk day 1	

### Measures

Primary outcome was post-operative LOS, which was measured as the number of hospital days between the patient’s admission on morning of surgery (Post-Operative Day, POD 0) to the discharge date. Secondary outcome measures included daily opioid consumption in morphine milligram equivalent (MMEs) and post-operative pain intensity scores. Morphine equivalents were calculated for each opioid used post operatively based on chart data. Pain intensity was measured using the numeric rating scale (NRS). ERAS protocol elements included POD of urinary catheter removal, POD of first mobilization out of bed, in addition to ITM use and NSAID use (Table 1). The total number of protocol elements that each patient received was calculated and ranged from 0 to 4. Other clinical variables obtained from chart review included age (in years), sex, weight, height, number of fused levels, and pre-and post-operative magnitude of the major coronal deformity represented by Cobb angle. Use of transfusion of red blood cells, other surgical complications and return to hospitalization within 30 days post-surgery were also assessed.

### Statistical analysis

Sample characteristics were summarized using means and standard deviations for continuous variables and frequency counts and percentages for categorical variables and compared by protocol using independent *t* test and chi squares tests, as appropriate. Prevalence of use of each protocol element was calculated along with the proportion that received 0, 1, 2, 3 or all 4 protocol elements. Median LOS was estimated and compared by protocol type using a Mann–Whitney test. Median LOS was also summarized and compared according to protocol element and number of protocol elements received using a Kruskal–Wallis test with pairwise comparisons and p value adjusted for multiple comparisons.

To address the primary objective, differences in LOS according to protocol were assessed using regression to allow for adjustment of age, sex, BMI, pre-surgical Cobb angle, levels fused, use of blood transfusion and year of surgery. The latter was adjusted to account for potential improvements in protocol implementation over time. LOS is a count of the number of events (i.e., hospital days) that occurred within a defined time period. Counts are a set of non-negative integers that have specific distributional properties, which are best approximated by Poisson or negative binomial distributions [12]. Extensions of these models have been proposed to accommodate count distributions that contain excess zeros (inflated) or lack of zeros altogether (truncated). In this case, since hospitalization occurs at the time of surgery, a zero count is not possible and use of a zero-truncated model is recommended to avoid bias, which increases as the mean count becomes closer to zero. A zero-truncated Poisson model was run using the proc FMM in SAS to estimate adjusted Rate Ratios and 95% confidence intervals. As under-dispersion was found in the data (i.e., the variance was smaller than the mean), this model was compared with a generalized Poisson model, which can accommodate under-dispersion [13].

For the secondary outcomes of pain intensity scores and opioid use, repeated-measures analysis was undertaken using linear mixed models comparing the ERAS and N-ERAS groups. Time was modeled categorically to allow for a non-linear relationship between time and the outcomes. An unstructured correlation matrix was used to model random effects. The model included protocol, time, time by protocol interaction, and the control variables defined above as independent variables; the interaction was included to determine if the trajectories of pain/opioid use varied over time by protocol. The first model used average pain as the outcome, the second used opioid consumption (measured in morphine milligram equivalents (MME) per day), and the third, average pain while controlling for opioid consumption over time.

To address the final objective of determining predictors of LOS, two models were specified. The first model included each protocol element separately. The second model included the number of protocol elements received (entered as a categorical variable). Both models used a zero-truncated Poisson regression model as described above and controlled for age, sex, BMI, pre-surgical Cobb angle, levels fused, year of surgery and blood transfusion. Surgical complications (e.g., infections) and hospital readmission within 6 months of surgery was compared between the ERAS and N-ERAS groups using a Chi square test. All analyses were undertaken using SAS (v 9.4). A two-sided alpha of 0.05 was used to establish statistical significance (unless otherwise stated).

## Results

140 AIS patients met the study inclusion criteria, 59 were included in the ERAS group and 81 in the N-ERAS group. As shown in Table 2, baseline characteristics of the ERAS and N-ERAS groups were similar with respect to sex, BMI, pre-and post-operative Cobb angle, and levels fused. Patients in the ERAS group were younger on average (14.1, SD = 1.8) compared to the N-ERAS group (15.0, SD = 1.8; *p* = 0.008). Also, due to protocol implementation, more surgeries in the N-ERAS group took place earlier in the study period than the ERAS group (*p* = 0.025). Finally, more of those in the N-ERAS group required a transfusion of red blood cells than those in the ERAS group (*p* = 0.004).

**Table 2:** Characteristics of the sample according to protocol group

	N-ERAS (*n* = 81)	ERAS (*n* = 59)	*p* value*
Female sex	69 (85.2)	55 (93.2)	0.14
Age	15.0 (1.8)	14.1 (1.8)	0.008
BMI	20.5 (4.0)	20.3 (4.0)	0.705
Pre-op Cobb angle	74.6 (16.5)	69.9 (13.6)	0.08
Post-op Cobb angle	19.11 (10.7)	19.3 (7.0)	0.892
Levels fused	11.4 (1.8)	11.4 (1.7)	0.826
Year of surgery			
2018	15 (18.5)	3 (5.1)	0.025
2019	41 (50.6)	28 (47.5)	
2020	25 (30.9)	28 (47.5)	
Blood transfusion	37 (45.7)	13 (22.0)	0.004
Length of stay (hours)	120.8 (21.3)	92.7 (36.3)	< 0.0001

*p value compares characteristics of the N-ERAS and ERAS groups

All patients in the ERAS group were treated with ITM intra-operatively and with NSAIDs postoperatively and were discharge only after meeting a prespecified criteria checklist. 66% of patients were mobilized on POD 0 and 71% had their urinary catheter removed on POD 1. 58% of patients received all four protocol elements; 20% received two, and 22% received three.

Among those in the N-ERAS group, 26% had the urinary catheter removed on day 1, 2.5% had early mobilization, 7.4% received NSAIDS, none had ITM intra-operatively. In the N-ERAS group, 65.2% received none of the protocol elements, 33% received one, and 1.2% received two protocol elements (Table 3).

**Table 3:** Protocol specific characteristics

	N-ERAS (*n* = 81)	ERAS (*n* = 59)
Urinary catheter removal POD 1	21 (25.9)	42 (71.2)
Mobilization POD 0	2 (2.5)	39 (66.1)
Use of intrathecal morphine	0 (0.00)	59 (100.0)
Use of NSAIDS	6 (7.4)	59 (100.0)
Number of protocol elements received		
None	53 (65.4)	0 (0.0)
One	27 (33.3)	0 (0.0)
Two	1 (1.2)	12 (20.3)
Three	0 (0.0)	13 (22.0)
Four	0 (0.0)	34 (57.6)

### Length of stay

Median LOS was 3 days (IQR = 3–4) for the ERAS group, compared with 5 days (IQR = 4–5) for the N-ERAS group (*p* < 0.001). Each protocol element when studied separately significantly reduced LOS. In terms of LOS in hours there was on average 28.1 h reduction between the N-ERAS and ERAS groups (*P* < 0.0001). Early urinary catheter removal and use of NSAIDS each reduced LOS from a median of 5 days (IQR = 4–5) to a median of 3 days (IQR = 3–4, both *p* < 0.001). Use of ITM pre-operatively and early mobilization each reduced LOS from a median of 4 days (IQR = 4–5) to a median of 3 days (IQR = 3–4, both *p* < 0.001). Median LOS stay declined by the number of protocol elements received (zero: 5, IQR 4–5; one: 4, IQR 4–5; two: 4, IQR: 4–5; three: 3, IQR: 3–4; four: 3, IQR:3–3) *p* < 0.001). Using an adjusted p value of *p* = 0.005, median LOS was lowered significantly with three (*p* < 0.001) or four components (*p* < 0.001) compared to none, and four elements compared to one (*p* < 0.001) or two (*p* = 0.001).

**Fig. 1:**
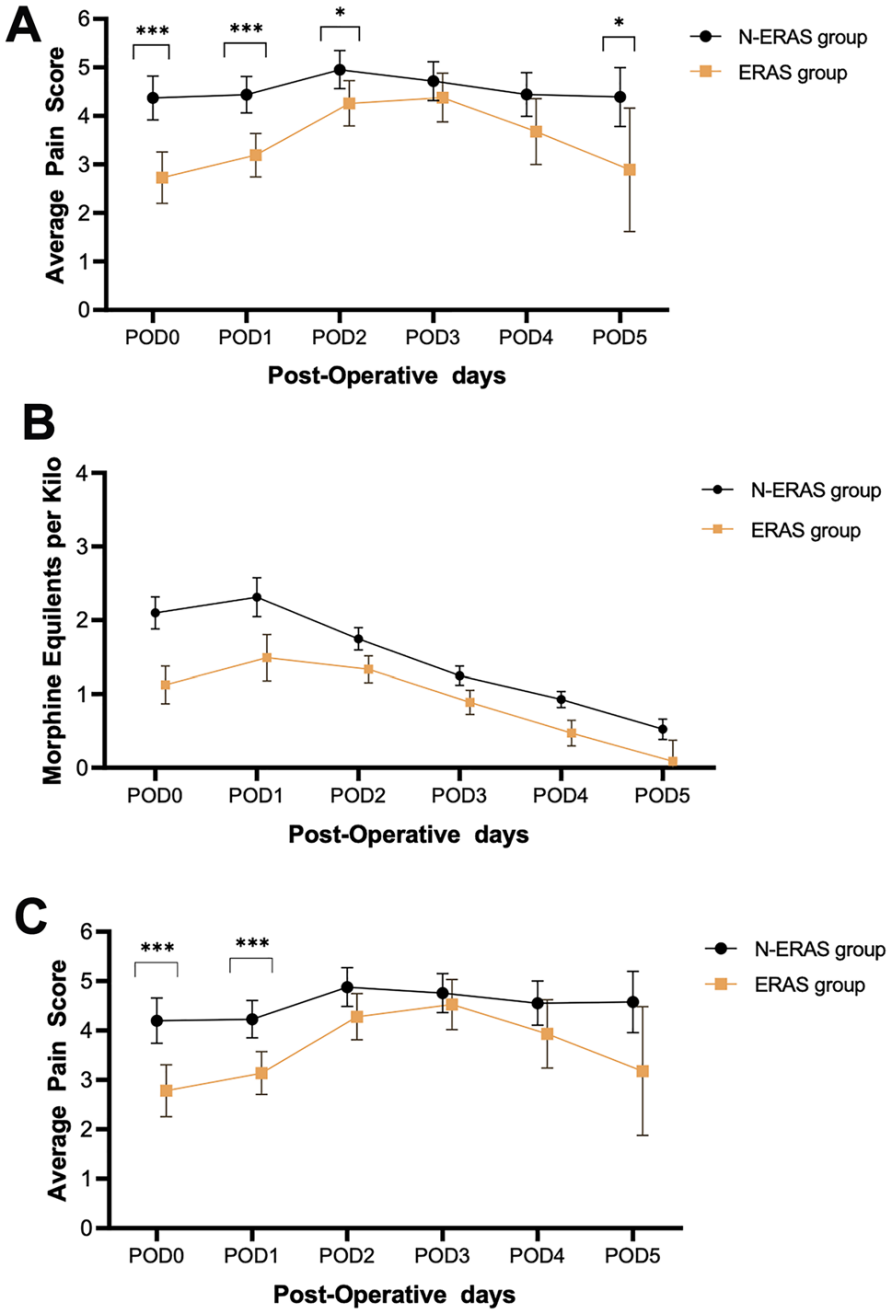
Zero-truncated poisson regression model of the effect of protocol on length of stay. *ERAS* Enhanced recovery after surgery group, *N-ERAS* standard care group. ^a^The rate ratio for ERAS vs. N-ERAS can be understood as those receiving the ERAS protocol stayed about 75.1% of the time as those receiving standard care

The model of the effect of protocol (ERAS vs. N-ERAS) on LOS using zero-truncated Poisson regression is presented in Fig. 1. After controlling for age, sex, BMI, pre-operative cobb angle, number of levels fused, year of surgery and blood transfusion, the ERAS group had a significantly shorter rate of stay. Specifically, those treated with the ERAS protocol stayed 75% of the time of those in the N-ERAS group (RR = 0.75; 95% CI = 0.62–0.92). Based on predicted values, this is equivalent to a median of 1.3 days shorter in the ERAS groups. This model was highly consistent with the generalized Poisson model (see Supplementary Figure).

### Pain and opioid use

**Fig. 2:**
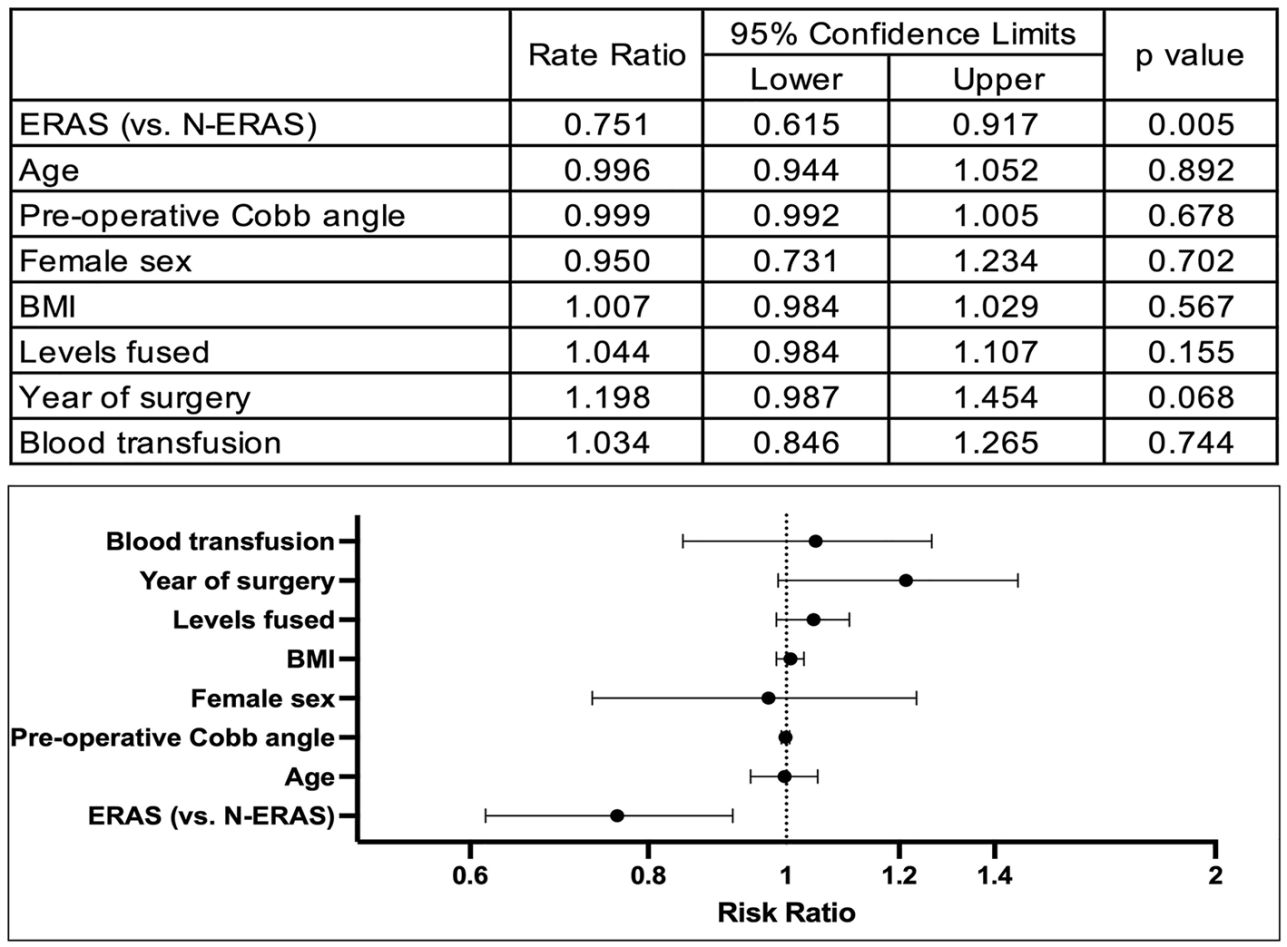
Post-operative pain and opioid utilization. **A** average pain scores per day (Least square means). **B** Opioid utilized per day in mg/kg. **C** Pain scores per day normalized by opioid doses. (***P < 0.001, *P < 0.05)

Figure 2A–C present the trajectories of pain, and opioid use for the ERAS and N-ERAS groups derived from the linear mixed models. The ERAS group was found to have significantly lower average pain on POD 0 (least square means [LSM] = 2.66 vs. 4.41, *p* < 0.001), POD 1 (LSM = 3.12 vs. 4.48, *p* < 0.001), POD 2 (LSM = 4.19 vs. 4.99, *p* = 0.013), POD4 (LSM = 3.61 vs. 4.48, *p* = 0.040) and POD 5 (LSM = 2.84 vs. 4.42, *p* = 0.035, see Fig. 2A). At each time point, the ERAS group had a significantly lower average daily MME (mg/kg) (POD 0: LSM = 1.11 vs. 2.11; POD 1: LSM = 1.47 vs. 2.32; POD 2: LSM = 1.32 vs. 1.75; POD 3: LSM = 0.87 vs. 1.26; POD 4: LSM = 0.46 vs. 0.94; POD 5: LSM = 0.09 vs. 0.54; all *p* < 0.01, see Fig. 2B). When looking at average pain intensity while controlling for average morphine equivalent dosage, significantly lower pain was observed in the ERAS group for POD 0 (LSM = 2.7 vs. 4.2, *p* < 0.001), POD 1 (LSM = 3.1 vs. 4.3, *p* < 0.001), POD 2 (LSM = 4.2 vs. 4.9, p = 0.028), and POD 5 (LSM = 3.1 vs. 4.6, *p* = 0.046, see Fig. 2C).

### Predictors of length of stay

**Fig. 3:**
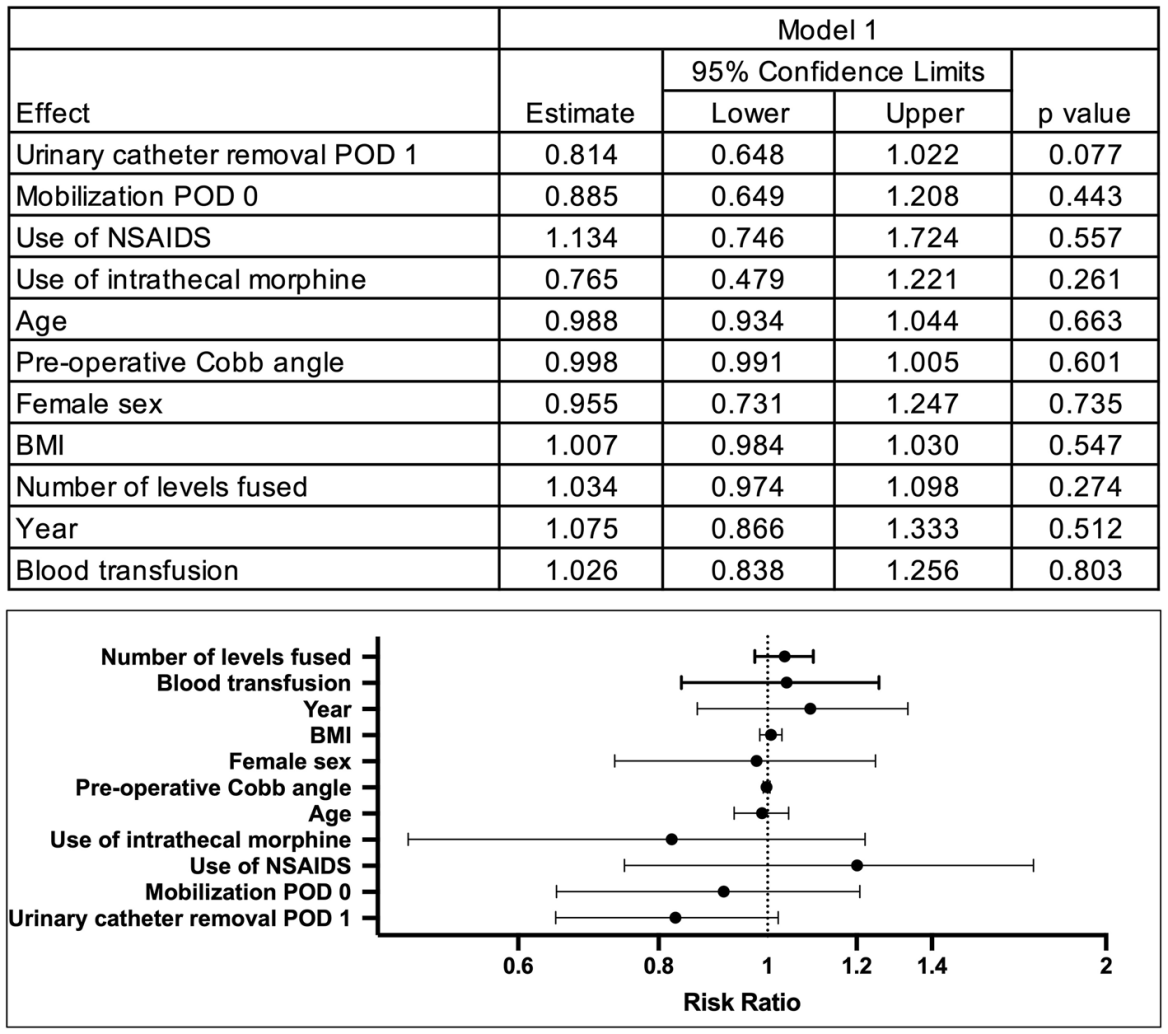
Zero-truncated poisson regression model of predictors of length of stay—model 1. *POD* Post-operative day, *BMI* Body mass index. ^a^The rate ratio for urinary catheter removal can be understood as those having had their catheter removed on POD 1 stayed about 81% of the time as those who had the catheter removed later

**Fig. 4:**
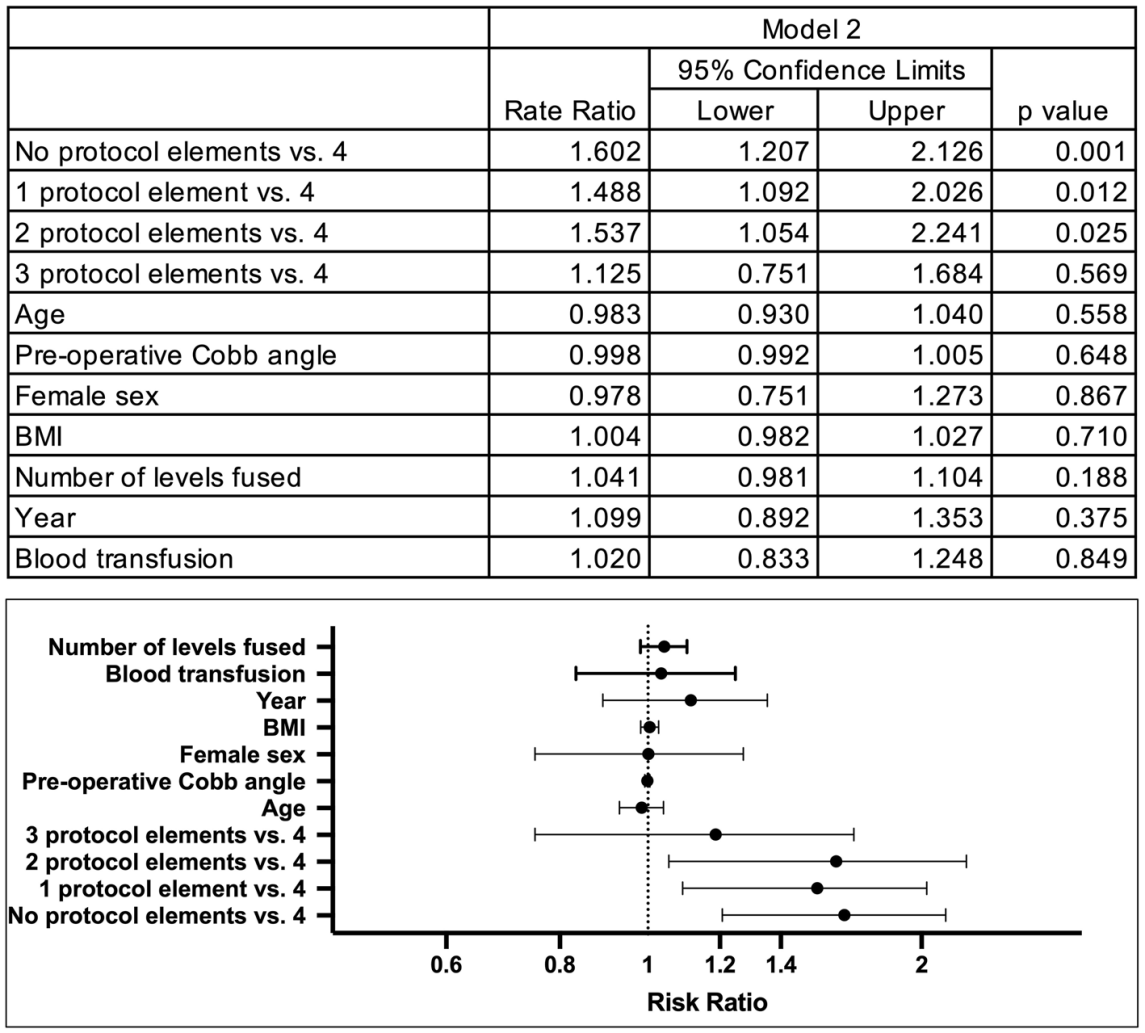
Zero-Truncated Poisson Regression Model of Predictors of Length of Stay—Model 2. *BMI* Body Mass Index. ^a^The rate ratio for no protocol elements vs. 4 can be understood as those receiving none of the ERAS protocol elements stayed about 60% greater the time as those who received all 4 ERAS protocol elements

Models of predictors of LOS are presented in Figs. 3 and 4. Model 1 uses each element of the protocol as predictors and Model 2 used the total number of protocol elements received. As shown, none of the individual protocol elements were associated with LOS over and above any other element, when controlling for age, sex, BMI, pre-operative Cobb angle, year of surgery and blood transfusion (see Fig. 3, Model 1). However, when looking at number of protocol elements received, a significant association was found. Compared to those receiving all four elements, those receiving two (RR = 1.54 95% CI = 1.05–2.24), one (RR = 1.49; 95% CI = 1.09–2.03) or none (RR = 1.60, 95% CI = 1.21–2.13) of the protocol elements had significantly longer stays after adjustment. Based on predicted values, these amount to median stays of 1.7, 1.3 and 1.8 days longer, respectively. No difference was found in LOS between those receiving three and four protocol elements (*p* = 0.569). When switching the reference group to receiving none of the protocol elements, significantly shorter stays were found for those receiving four (RR = 0.62, 95% CI = 0.47–0.83) elements. No differences were found between receiving three (*p* = 0.062), two (*p* = 0.788) or one element (*p* = 0.538) and receiving none.

### Complications and re-hospitalization

One patient in the ERAS group returned to the hospital on POD 13 due to grade 3 burns from a hot pack that was applied incorrectly. The patient was hospitalized and treated by plastic surgery with debridement and skin graft.

## Discussion

Implementation of modified ERAS-based protocol on patients with AIS undergoing PSF was associated with a shorter LOS, lower opioid consumption and lower average pain scores when compared to comparable groups of patients, treated at the same institution during the same time period, who received standard care (N-ERAS). Furthermore, we found evidence that each protocol element contributed to shortening LOS without clear superiority of one protocol element over another.

Originally, ERAS pathways were implemented in general surgery but have since grown in popularity in other surgical fields. The idea behind ERAS is that by protocolizing care in a way that prioritizes patients’ physiologic needs and integrating these priorities across the perioperative pathway, including all the relevant disciplines, creates a safer environment, reduces complications and improves outcomes [14]. A recent meta-analysis looking at 2456 patients with AIS treated surgically, found that patients treated by ERAS emphasized that protocol had a shorter length of stay, earlier ambulation, earlier removal of urinary catheters and earlier discontinuation of patient-controlled analgesia [15]. While adoption of all AIS relevant ERAS strategies might be challenging, in our study we were able to model separate protocol elements and look at their individual contribution to the primary outcome.

LOS is an important outcome measure when analyzing the impact of ERAS initiatives. LOS is important for two reasons, firstly, each additional hospital day represents a significant resource utilization with its associated costs. Interventions that succeed at reducing the cost needed to treat, are compelling for adoption by the payers at all levels. Most notably, in a resource constrained setting, this savings leads to an increased capacity to care for other patients. Secondly, and more importantly, a reduced hospital LOS likely results from an overall accelerated recovery across several domains (pain control, ability to ambulate, ability to self-care, adequate education for coping at home). It is therefore a convenient, objective, measurable surrogate for the overall quality of care and subsequent recovery.

In our study, ERAS participants stayed 75% of the time of those receiving standard care, when adjusting for a variety of variables, including pre-surgical cobb angle. A reduction of 20–48% in the length of stay has been previously reported by other authors [16, 17]. This heterogeneity in LOS reduction might be attributed to different reporting definition of LOS. Nevertheless, it is clear that pathways committed to ERAS methodology led to shorten LOS.

Pain control and opioid reduction are thought to be imperative for a successful implementation of an ERAS protocol. Multimodal analgesia is fundamental to not only optimize pain control but also minimize side effects by reducing opioid consumption. We were able to demonstrate lower average pain scores through the post-operative period with reduction of 1.65 NRS points on the day of surgery and by 1.2 points on the first post- operative day while considerably reducing opioid requirements. These differences were both statistically and clinically significant. The addition of ITM and routine use of NSAIDs was associated with a reduction of overall opioid usage.

Only few studies addressed pain management in the context of AIS and ERAS or RRP. Sanders et al. [16]. Reported on mild but statistically significant increase in pain scores beginning at POD 1 among those patients that were treated with rapid recovery pathway. It is not clear from this work how pain was addressed once PCA duration was shortened. Gornitzky et al. presented reduction in pain scores for days 0–2 following PSF among patients treated with a rapid recovery pathway [6]. Adding gabapentin and acetaminophen before surgery, intraoperative methadone and shortening IV PCA treatments were effective in reducing post-operative average pain on day 0 but the clinical significance might be questionable (VAS 3.9 ± 2 vs. 4.6 ± 2.1) as the minimal clinical important difference is most often considered to be 1 or more [18]. Nevertheless, pain reduction on days 1 and 2 were both statistically and clinically significant. Furthermore, they reported on significant reduction in the total opioids used on the day of surgery with no difference afterwards.

Muhly et al. [7] demonstrated that through longitudinal intervention and ERAS implementation post-operative pain scores on day 0 through 2 after surgery were reduced. In their initiative acetaminophen, gabapentin and ketorolac were introduced together with shortening of PCA duration.

Finally, Julien-Marsollier et al. [19], reported their ERAS implementation results. Pain management among all patients included ITM, gabapentin, acetaminophen, and NSAIDs. ERAS patients did not get any background morphine with the PCA and transition to oral analgesics was rapid combined with accelerated regain of oral intake. That resulted in significant reduction in pain scores and overall opioid consumption. Regardless the exact methodology, all ERAS initiatives are linked by acknowledging that pain management is crucial for a successful post-operative patient journey.

Reducing overall opioid consumption is critical for ERAS or rapid recovery initiatives to be successful. Nevertheless, probably due to the retrospective nature, only few studies completed analysis of opioids consumed post operatively and evaluated the effect of reduced opioid utilization. With a combination of ITM morphine prior to surgery and post-operative utilization of NSAIDs either intravenous or oral when tolerated, we were able to reduce the absolute opioid doses consumed by 1 mg/kg on POD0 and by 0.8 mg/kg on POD1.

Gornitzky et al. [6] reported that implementation of rapid recovery pathway resulted in 24% reduction of opioids utilization on POD 0 but no differences were recorded on days 1 and 2 post-surgery. Interestingly, compared to the control group, the majority of the patients were treated with oral analgesics rather than with IV PCA. Once again, the authors demonstrated significant reduction in LOS that couldn’t be directly corelated to opioid reduction.

It is clear that regardless of the strategy used, shortening the period of intravenous PCA, and thereby the requirement to maintain IV access, eliminating background doses of PCA, using ITM during the surgical intervention, early transition to oral opioids, incorporating more non-opioid medications and overall reduction of total amounts of opioid delivered lead to shortened LOS, better pain control and reduction of opioids and their side effects [8, 15].

Implementation of a new protocol is challenging and therefore we started with elements that we thought to be easy to apply with the largest potential impact aspiring to include all. We began by implementing four core elements (modified ERAS). Looking into adherence rates, 100% of the included patients were treated with intraoperative ITM, post-operative NSAIDs (those were inclusion criteria) and discharge check list, but only 66% of our ERAS patients were mobilized on day 0 and in 71% urinary catheter was removed on Day 1. Interestingly, we could not find a single protocol element that could predict shorter LOS over and above all other elements. On the contrary, it was the combination of several elements that was associated with shorter LOS. In particular, receiving 3 or more elements seemed to be the critical number to affect LOS, which is suggestive of a threshold effect.

The importance of adherence to ERAS components was previously demonstrated in a nationwide study on a large number of adults treated with posterior instrumented fusion of the lumbar spine (4). Among the screened population treated between the 2006–2016, high utilization of ERAS (more than five agreed protocol elements) compared to low utilization (less than three agreed elements) was associated with reduced LOS, lower complication rates and some cost reduction.

In our study a single patient in the ERAS group was readmitted for surgical treatment by a plastic surgery team due to severe skin burn caused by hot pack misuse. No other complications were recorded in either of the groups and the single readmission was not related to the new protocol.

There are limitations to our study. The patients treated with the ERAS protocol where all treated by the same surgeon. Patient falling in the N-ERAS group were treated by two other surgeons. Previous studies have demonstrated that surgeon-specific factors can impact patient outcomes, including post-operative complications, length of hospital stay, and readmission rates. Furthermore, surgeon volume, experience, training, and proficiency in a particular technique can all impact pot-operative outcomes. Therefore, outcomes might be influenced by the surgical technique and the individual surgeon. While we did not directly assess the impact of surgeon on our outcomes, we acknowledge that this is an important factor that could have influenced our findings. However, we attempted to mitigate this by providing clear guidelines for the N-ERAS classification and standardizing pre-operative and post-operative care. Nonetheless this remains a limitation of the study.

Secondly, partial adherence to the protocol in our cohort, can be contributed to two major components; an initial learning curve, which once resolved led to improved adherence overtime and some staff resistance to change due to affinity for status quo practice culture. For these reasons, we observed that providers, including, surgeons, nurses, and physiotherapists used to N-ERAS protocol had some difficulties applying the new modified ERAS protocol to patients under their care. Third, differences in age between the groups that were significant on univariate analysis, and this could theoretically contribute to some of the differences in the unadjusted results. Nevertheless, knowing that early removal of urinary caterers, better pain management on the day of surgery represented by better pain scores and lower opioid consumption, and early ambulation suggests that in spite of the limitations mentioned above the primary and secondary outcomes were significantly better. Furthermore, we now better understand the individual contribution of each protocol element and even partial inclusion of some of the ERAS protocol elements might result in significant gains and better quality of care.

## Conclusion

Based on our cohort, a modified ERAS intervention for AIS patients results in significantly shorter LOS after surgery, reduced pain, and lower opioid consumption. The use of intrathecal morphine, NSAIDs, early removal of urinary catheter, and early ambulation when combined contributed to shorter LOS. Implementation of ERAS principles and better adherence to the suggested protocol might promote further change in the LOS, pain control and opioid consumption.

## Supplementary Information

Below is the link to the electronic supplementary material.

Supplementary Generalized Poisson Regression Model of the Effect of Protocol on Length of Stay. file1 (JPG 412 KB)

## Data Availability

Not applicable.
