# Supplementary figures and images for: Utilization of individual components of enhanced recovery after surgery (ERAS) protocol improves post-operative outcomes in adolescent idiopathic scoliosis: a blueprint for progressive adoption of ERAS
Source: Spine Deform. 2023 May 26;11(5):1117–25. doi: 10.1007/s43390-023-00706-w (PMC10425294; doi:10.1007/s43390-023-00706-w)

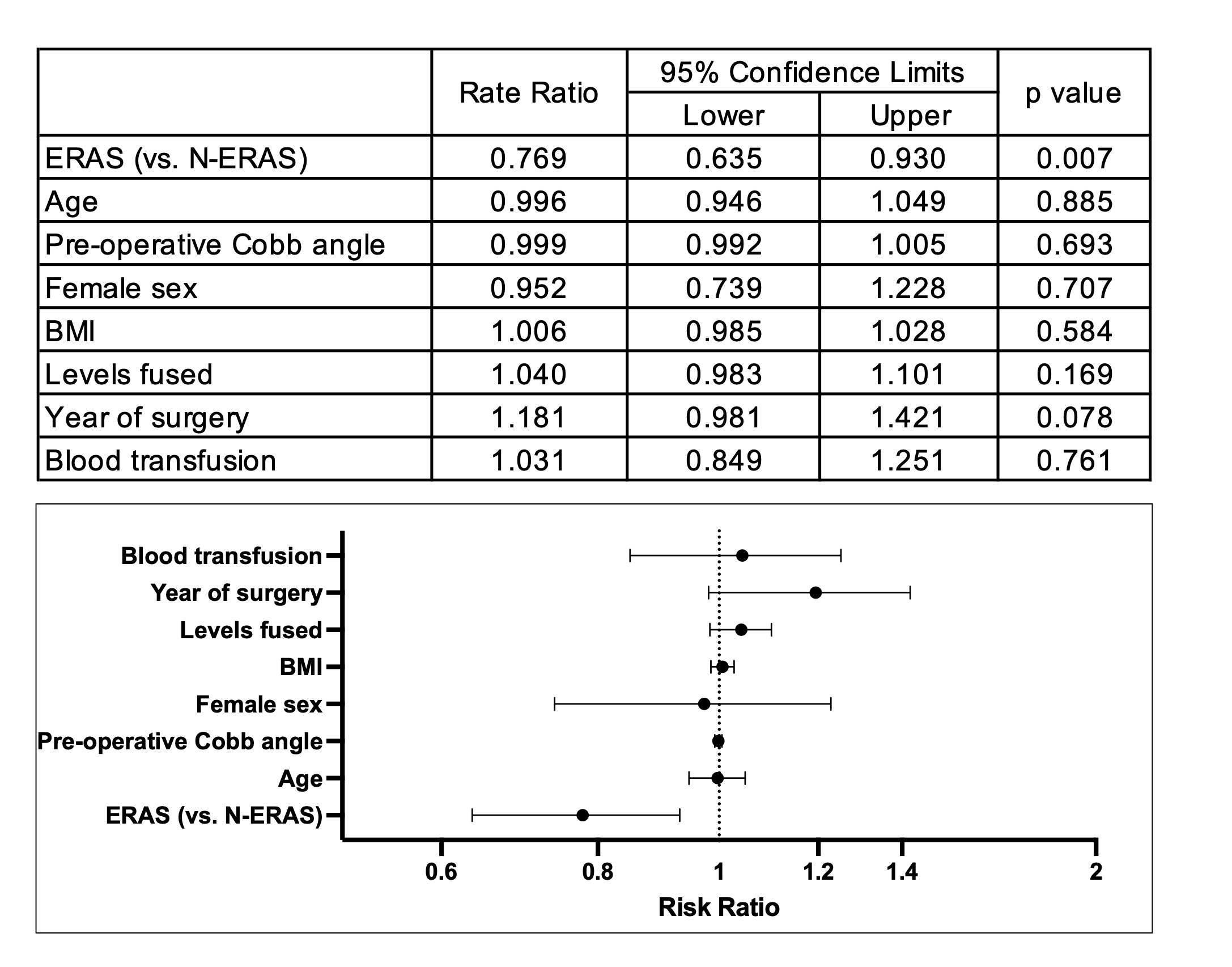

Supplement: Supplementary file 1 — Supplementary Generalized Poisson Regression Model of the Effect of Protocol on Length of Stay. file1 (JPG 412 KB) [file 43390_2023_706_MOESM1_ESM.jpg]
